# Primary pulmonary lymphoepithelioma-like carcinoma initially diagnosed as squamous metaplasia: A case report and literature review

**DOI:** 10.3892/ol.2015.2975

**Published:** 2015-02-17

**Authors:** YASHA LIANG, CHENG SHEN, GUOWEI CHE, FENGMING LUO

**Affiliations:** 1 Department of General Practice, West-China Hospital, Sichuan University, Chengdu, Sichuan 610041, P.R. China; 2 Department of Cardiovascular and Thoracic Surgery, West-China Hospital, Sichuan University, Chengdu, Sichuan 610041, P.R. China

**Keywords:** lymphoepithelioma-like carcinoma, pulmonary

## Abstract

A mass was detected in the middle lobe of the right lung of a 58-year-old female. The patient did not present any symptoms and was a nonsmoker. Diagnostic evaluation revealed squamous metaplasia in the middle lobe of the right lung. During surgery, a tumor was identified, which was diagnosed as a lymphoepithelioma-like carcinoma (LELC). LELCs have been mainly reported in the Asian population and are associated with the Epstein-Barr virus (EBVs), while they are not associated with smoking. Squamous metaplasia, which is the basis of squamous cell carcinoma, differs from LELC in the therapeutic methods used and the prognostic evaluation. Squamous metaplasia requires regular follow-up in out-patient clinics, while pulmonary LELC is treated by surgery and chemotherapy. Therefore, distinguishing between LELCs and other nonmalignant or premalignant conditions is essential.

## Introduction

Lymphoepithelial carcinoma is a nasopharyngeal carcinoma with lymphoid stroma and nonkeratinizing squamous cells. Lymphoepithelioma-like carcinomas (LELCs) arise on the exterior of the nasopharynx; however, they resemble lymphoepithelial carcinomas histologically. LELCs commonly occur close to the nasopharynx, while they have also been detected in other sites, including the salivary glands (1), lungs (2–4), skin (5), liver, cervix, urinary bladder (6), breast (7), thymus and stomach (8). Certain LELC types are associated with Epstein-Barr virus (EBV) infection (particularly LELCs of the stomach, salivary glands, lungs, skin and thymus) (4,5,9). Pulmonary LELCs are rare malignancies, usually detected in nonsmokers (10–13). A total of 9,851 patients with NSCLC were identified. Among these patients, 37 (0.4%) were diagnosed with lung LELC. These 37 patients were all from Southern China (14). Chang et al (11) estimated that pulmonary LELC represents ~0.92% of all lung cancers, further illustrating the rarity of pulmonary LELCs. Primary pulmonary LELC exhibits no significant gender predisposition and a minimal association with smoking history, however, it exhibits a strong association with EBV in Asian populations, and a predisposition for early or locally advanced stages of the disease. In a previous study, the mean age of patients with lung LELC was reported to be 10 years younger than that of patients with other histological types of lung carcinoma (14). Currently, the youngest pulmonary LELC patient reported in the literature is an eight-year-old child (15). The majority of patients undergo complete resection, as well as chemotherapy and radiotherapy for the treatment of pulmonary LELC. Recently, a study of 52 primary pulmonary LELC patients demonstrated that the two- and five-year overall survival rates were 88 and 62%, respectively, with the majority of patients diagnosed at early or locally advanced stages of the disease (16). The present study investigated the case of a female nonsmoker with pulmonary LELC. Written informed consent was obtained from the patient.

## Case report

A mass was detected on the middle lobe of the right lung of a 58-year-old female, during a medical check-up at the West-China Hospital (Chengdu, China) in January 2013. The patient was asymptomatic and physical examination identified no positive findings. The female had no history of smoking and alcohol use.

**Figure 1:**
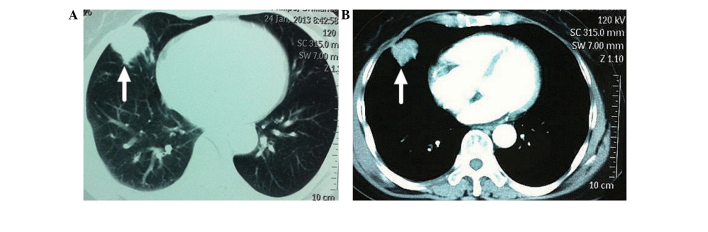
Computed tomography (CT) scans revealing a tissue mass. Enhancement thoracic CT demonstrated a mass in (A) the middle lobe of the right lung and the (B) mediastinum window, which was a possible lung tumor.

Enhancement computed tomography (CT) of the thorax revealed a mass in the middle lobe of the right lung, which was considered to be a possible lung tumor (Fig. 1). In addition, another small lesion was detected in the same lung lobe; however, this was not considered to be a metastatic lesion. Fibrobronchoscopic brushing (17) demonstrated the presence of squamous metaplasia with severe hyperplasia at the middle lobe of the right lung. A bone scan and a CT scan of the skull indicated no metastasis.

**Figure 2:**
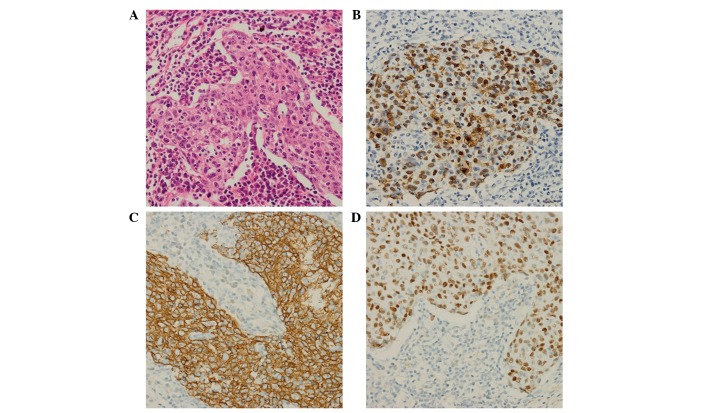
Histological features of lymphoepithelioma-like carcinoma of the lung. (A) Histological examination revealed a large island of tumor cells infiltrated by intense lymphoplasmacytic cell population (HE; magnification, ×400). (B) Cells were positive for Epstein-Barr virus (EBV)-encoded small nonpolyadenylated RNA (magnification, ×400). Immunohistochemical staining revealed that the specimen cells were positive for (C) cytokeratin 5/6 (magnification, 400x) and (D) P63 (magnification, ×400). HE, hematoxylin and eosin.

Surgery was performed under induction with midazolam (0.05–0.1 mg/kg) followed by the subsequent use of intravenous anesthesia (2 μg/kg, sufentanil; 2 mg/kg/h, propofol) with tracheal intubation. The patient underwent a lobectomy of the middle lobe (including sequential resection of the right pulmonary middle lobe vein, artery and trachea) and systematic mediastinal lymphadenectomy (group 2–4, 7 and 9–11 lymph nodes were swollen). The resected tissue and lymph nodes were frozen and biopsy was performed, revealing evidence of carcinoma. The surgery was completed following careful hemostasis and washing of the pleural cavity with warm saline solution. The patient did not present any complications, such as cough, chest pain and hemoptysis. No adjuvant chemotherapy and radiotherapy were performed. The patient was discharged a week after surgery and follow-up visits were scheduled. The resected specimen was 10×5×3 cm in size, containing a 3×2.5×2 cm tumor. Histologically, the tumor was solid and off-white in color, with a clear demarcation between the surrounding normal lung tissues, while pleural invasion was observed. Immunohistochemical analysis revealed that the tumor cells were positive for protein kinase C, p63, cytokeratin 5/6 (CK 5/6) and EBV-encoded small RNA (EBER), whereas the cells were negative for CK 7 and thyroid transcription factor-1 (Fig. 2). Lymph nodes collected during the surgery revealed no metastasis. Furthermore, the histological and immunohistochemical analyses confirmed the diagnosis of pulmonary LELC. The patient was healthy and asymptomatic following surgery. Thoracic enhancement CT revealed no signs of metastasis at three, six and 23 months following surgery.

## Discussion

A network database search of PubMed and Web of Science was conducted using the keywords “pulmonary” and “LELC” for studies reported in the English language between 1987 and 2015. A total of 196 such cases (male, 96; female, 100) were described in the literature (Table I), and patient age ranged between 8 and 83 years. Among the 196 patients, 111 were smokers (56.63%) and 45 were non-smokers (22.96%), however, information regarding smoking status was unavailable for 40 (20.41%) patients. The first pulmonary LELC case was described by Bégin *et al* in 1987 (4). Of the 196 cases reported, the majority of cases involved Asian patients, with approximately two-thirds of cases arising in southern China (12,40), Taiwan (11) and Hong Kong (29), illustrating the geographical distribution characteristics of pulmonary LELC. A close association exists between pulmonary LELCs and EBV infection, which is absent in other types of lung carcinomas. Among the 196 patients reported in the literature (Table I), 145 patients (73.98%) tested positive for EBV infection, 42 patients (21.43%) patients tested negative for EBV infection and information was unavailable for nine patients (4.59%). Previous studies have identified the presence of EBV infection in the tumor cells or serum of LELC patients (19,26,41). Circulating serum EBV DNA may be used as a tumor marker in the clinical management of patients with lung LELC (9,11,40,42). A study demonstrated that patients with a pretherapy serum EBV DNA level of >10,000 copies/ml exhibited significantly lower overall survival rates (18). Accurate diagnosis is significant and a prerequisite for treatment.

The diagnosis of lung LELC is usually based on the results of cytopathologic, histopathologic, immunohistochemical and EBER-positivity analyses, as well as a detailed systemic examination to exclude a possible extrapulmonary (nasopharyngeal) origin of the carcinoma and other lung diseases (43). Imaging diagnostic methods, including CT or magnetic resonance imaging (MRI) scans, are able to identify nonspecific lesions that resemble other pulmonary carcinomas. On CT scans, pulmonary LELCs usually appear as large, central, well-defined and lobulated tumors with vascular or bronchial encasement and obstructive pneumonia (43). Calcification has been rarely observed in pulmonary LELCs. In addition, MRI scans of LELCs usually detect an isointense or low-intensity signal on T1-weighted images and a slightly increased signal on T2-weighted images, while enhancement of abnormal tissue is typically observed (19,44). The cytological features of the specimens are commonly analyzed by needle aspiration or fibrobronchoscopic brushing, which reveal abnormal cell morphology that usually appears as large clusters of neoplastic cells with scant cytoplasm. The nuclei are normally large and hyperchromatic, with irregular contour and prominent nucleoli (20). Histologically, the tumors appear solid and off-white in color, with a clear demarcation between the surrounding normal pulmonary tissues, while occasionally pleural invasion is observed. Immunohistochemical analysis of pulmonary LELCs usually detects positive staining of membrane tumor markers, including latent membrane protein-1, viral capsid antigen and CKs (20). In addition, EBER detection is significant in the diagnosis of pulmonary LELCs, since EBER is absent in other lung carcinomas, such as non-small-cell lung carcinomas. Similar to nasopharyngeal carcinomas, pulmonary LELCs are sensitive to chemotherapy and radiotherapy (13,31). In early-stage pulmonary LELCs, the main treatment method is surgical resection, while comprehensive treatment (surgery, chemotherapy and radiotherapy) is adopted in patients with advanced or unresectable tumors (31). Previous studies have revealed that early-stage pulmonary LELC cases present an improved prognosis compared with advanced cases or other pulmonary carcinoma types in follow-ups after surgery (10,16).

Fibrobronchoscopic brushing is the most widely used method with a decisive role in the diagnosis of lung carcinomas. In the present study, fibrobronchoscopic brushing revealed squamous metaplasia with severe hyperplasia at the middle lobe of the right lung. However, immunohistochemical analysis diagnosed the presence of a pulmonary LELC. Squamous metaplasia, which is the basis of squamous cell carcinomas, differs from pulmonary LELC in the therapeutic methods used and the prognostic evaluation. Squamous metaplasia requires regular follow-up in out-patient clinics, while pulmonary LELC is treated by surgery and chemotherapy. Therefore, distinguishing between LELC and other nonmalignant or premalignant conditions is essential. The present study indicated that despite the rarity of pulmonary LELC, it should be included as one of the differential diagnoses for lung malignancies. Therefore, physicians must consider performing larger biopsies, particularly when histological examination of tissue removed during surgery remains unidentified.

## Figures and Tables

**Table I:** Patient characteristics of the 196 cases of pulmonary lymphoepithelioma-like carcinoma published between 1987 and 2015 in the English literature.

Author, year (ref)	Cases, n (F/M)	Age (years)	Smoking status, n (S/NS)	EBV, n (+/−)
Ma *et al*, 2013 (19)	41 (19/22)	25–74^a^	10/31	37/4
Jeong *et al*, 2013 (21)	1 (1/0)	60	0/1	1/0
Dong *et al,* 2015 (22)	1 (0/1)	83	N/A	N/A
Yener *et al*, 2012 (23)	1 (0/1)	62	1/0	0/1
Tanaka *et al*, 2012 (24)	1 (1/0)	71	0/1	0/1
Shen *et al*, 2012 (25)	1 (1/0)	75	1/0	1/0
Hayashi *et al*, 2012 (20)	1 (0/1)	70	0/1	1/0
Xia *et al*, 2009 (26)	21 (8/13)	40–67^a^	7/14	12/9
Bildirici *et al*, 2005 (27)	1 (1/0)	66	0/1	0/1
Ngan *et al,* 2004 (18)	19 (10/9)	52.7^b^	8/11	11/8
Kobayashi *et al,* 2004 (28)	1 (1/0)	67	N/A	1/0
Ho *et al*, 2004 (29)	10 (5/5)	38–71^a^	2/8	6/4
Hernández Vázquez, *et al* 2004 (30)	1 (0/1)	59	1/0	N/A
Abe *et al,* 2004 (31)	1 (1/0)	57	0/1	N/A
Morbini *et al,* 2003 (32)	1 (0/1)	25	0/1	1/0
Chang *et al,* 2002 (11)	23 (16/7)	42–80^a^	6/17	23/0
Han *et al,* 2001 (12)	32 (10/22)	39–73^a^	N/A	30/2
Barroso *et al,* 2000 (33)	1 (0/1)	25	0/1	1/0
Kasai *et al,* 1999 (34)	1 (1/0)	39	1/0	1/0
Chen *et al,* 1998 (14)	5 (3/2)	43–66^a^	0/5	5/0
Wöckel *et al*, 1997 (35)	2 (1/1)	49, 66	N/A	N/A
Curcio *et al,* 1997 (15)	1 (1/0)	8	0/1	1/0
Wong *et al,* 1995 (36)	9 (1/8)	33–71^a^	4/5	9/0
Wöckel *et al,* 1995 (37)	1 (1/0)	47	0/1	NA
Higashiyama *et al,* 1995 (9)	2 (0/2)	55, 65	N/A	2/0
Ferrara and Nappi, 1995 (2)	2 (1/1)	64, 78	1/1	0/2
Chow *et al,* 1995 (38)	2 (0/2)	56, 66	N/A	N/A
Chan *et al,* 1995 (3)	11 (5/6)	38–73^a^	2/9	11/0
Miller *et al,* 1991 (39)	1 (1/0)	65	1/0	0/1
Bégin *et al,* 1987 (4)	1 (1/0)	40	0/1	N/A

a Age range.

b Mean age.

F, female; M, male; S, smoker; NS, non-smoker; EBV, Epstein-Barr Virus; +, EBV positive; −, EBV negative; N/A, data not available.
